# Chinese Yam Polysaccharides Alleviate Myocardial Ischemia/Reperfusion Injury by Modulating Gut Microbiota, Restoring Mitochondrial Function, and Reducing Oxidative Stress

**DOI:** 10.3390/nu18152464

**Published:** 2026-07-28

**Authors:** Zhengyang Zhang, Yang Zhang, Yufang Shi, Xinyu Luo, Zhixi Wei, Peng An, Yongting Luo, Junjie Luo

**Affiliations:** Department of Nutrition and Health, China Agricultural University, Beijing 100193, China; zzy2580a@cau.edu.cn (Z.Z.); sy20253314223@cau.edu.cn (Y.Z.); syff@cau.edu.cn (Y.S.); b20253311588@cau.edu.cn (X.L.); wzx10001000@cau.edu.cn (Z.W.)

**Keywords:** Chinese yam polysaccharides, myocardial ischemia/reperfusion injury, oxidative stress, inflammatory response, gut microbiota, cardioprotection

## Abstract

**Background/Objectives:** Myocardial ischemia/reperfusion (I/R) injury remains a critical challenge in cardiovascular disease management. Although Chinese yam polysaccharides (CYPs), the primary bioactive macromolecules isolated from *Dioscorea opposita* Thunb, exhibit well-documented antioxidant and anti-inflammatory properties, their cardioprotective efficacy against acute I/R injury and the underlying multiscale mechanisms remain unexplored. This study investigated the protective effects of CYPs using an in vivo mouse model of myocardial I/R injury. **Methods:** An in vivo mouse model of myocardial I/R injury was used to evaluate the effects of 7-day prophylactic CYPs treatment (400 mg/kg). Echocardiographic and histological analyses were performed, and serum myocardial injury biomarkers, oxidative stress indicators, pro-inflammatory cytokines, mitochondrial ultrastructure, ATP bioenergetics, mitochondrial respiratory chain gene expression, and gut microbiota composition were assessed. **Results:** Echocardiographic and histological analyses revealed that CYPs pretreatment significantly ameliorated cardiac dysfunction, as indicated by increased LVEF from 28.98% to 57.68% and reduced myocardial infarct size by 36.73% compared with the I/R group and decreased serum myocardial injury biomarkers, including CK-MB, LDH, and LDH-1. Mechanistically, CYPs exerted robust cardioprotection by mitigating oxidative damage, with MDA levels reduced by 28.83% and SOD activity increased to 1.76-fold that of the I/R group, and suppressing the release of pro-inflammatory cytokines, including *Tnf-α*, *Il-6*, and *Il-1β*. Crucially, CYPs intervention preserved mitochondrial ultrastructure and ATP bioenergetics, and levels increased to 1.51-fold that of the I/R group and upregulated the expression of essential mitochondrial respiratory chain genes, including *mt-Nd1*, *mt-Nd4l*, *mt-Cyb*, *mt-CoII*, and *mt-Atp6*. Furthermore, 16S rRNA sequencing showed that CYPs treatment reshaped gut microbiota and elevated the relative abundance of anti-inflammatory and antioxidant beneficial genus *Akkermansia*. **Conclusions:** Collectively, these findings provide novel evidence that CYPs confer profound protection against myocardial I/R injury through a multitargeted network involving the restoration of mitochondrial homeostasis, attenuation of oxidative inflammation, and modulation of the gut microbiome, highlighting CYPs as a promising functional food-derived candidate for adjunctive therapy in ischemic heart disease.

## 1. Introduction

Although reperfusion therapy has markedly improved short-term survival in patients with acute myocardial infarction (AMI), ischemia/reperfusion (I/R) injury remains a major obstacle to favorable long-term clinical outcomes. This pathological process is characterized by a complex interplay of oxidative stress, calcium overload, inflammatory responses, and multiple cell death pathways, resulting not only in myocardial stunning and microvascular dysfunction but also in irreversible myocardial injury. Importantly, I/R injury is a key driver of pathological ventricular remodeling and ultimately contributes to the progression to heart failure [1]. Therefore, multitarget interventions directed at the interconnected pathological networks of inflammation, oxidative stress, and cell death in I/R injury have emerged as a promising strategy for improving patient prognosis. In recent years, natural products with anti-inflammatory, antioxidant, and multipathway regulatory properties have attracted considerable attention [2,3]. Their intrinsically low toxicity and potential to exert synergistic effects on multiple targets provide a promising approach for addressing this complex pathological process.

*Dioscorea* spp., commonly known as Chinese yam, is a traditional medicinal and edible plant whose tubers are rich in a variety of bioactive constituents, including polysaccharides, dioscin, and allantoin [4]. Among these components, Chinese yam polysaccharides (CYPs) are regarded as one of its principal active constituents, and modern pharmacological studies have demonstrated that they possess a broad range of biological activities [5]. Although numerous studies have documented the diverse effects of CYPs on antioxidant activity, anti-inflammatory responses, and metabolic regulation, their specific role and underlying mechanisms in acute cardiovascular events, particularly myocardial I/R injury, remain unclear. Notably, the central pathological features of myocardial I/R injury—including oxidative burst, uncontrolled inflammation, activation of multiple programmed cell death pathways, and microvascular dysfunction—closely correspond to the established pharmacological targets of CYPs. This mechanistic overlap suggests that CYPs may confer cardioprotective effects by synergistically modulating the complex pathological network of I/R injury through their intrinsic multitarget properties. However, this hypothesis still requires experimental validation.

The gut microbiota is closely associated with myocardial I/R injury [6]. Certain microbial metabolites, such as trimethylamine N-oxide (TMAO), have been shown to aggravate cardiomyocyte ferroptosis and thereby exacerbate I/R injury [7]. In contrast, high-fiber diets promote the expansion of beneficial bacteria and increase the production of short-chain fatty acids, such as acetate, which confer cardioprotective effects by improving mitochondrial function and suppressing inflammatory responses [8,9]. Likewise, interventions involving probiotics such as Lactobacillus reuteri or its metabolite GABA, as well as bioactive compounds derived from traditional Chinese medicine such as flavin mononucleotide (FMN), have been reported to attenuate myocardial I/R injury by remodeling the gut microbial composition and inhibiting inflammatory pathways, including the ROS-TXNIP-NLRP3 axis [10,11]. Collectively, these findings support the rationale for targeting the gut microbiota as a therapeutic strategy for myocardial I/R injury. However, whether CYPs supplementation influences myocardial I/R injury through modulation of the gut microbiota remains to be determined.

In this study, we investigated whether CYPs could attenuate myocardial I/R injury by modulating gut microbial diversity and composition. Using a mouse model of myocardial I/R, we comprehensively evaluated the cardioprotective effects of CYPs by assessing cardiac function, myocardial infarct size, serum markers of myocardial injury, and levels of oxidative stress and inflammation in myocardial tissue. In parallel, we explored the underlying mechanisms.

## 2. Materials and Methods

### 2.1. Animals and Reagents

Male C57BL/6 mice (six-week-old) were obtained from Beijing Vital River Laboratory Animal Technology Co., Ltd. (Beijing, China). The animals were maintained under specific pathogen-free conditions with controlled temperature (20–26 °C), humidity (40–70%), pressure (45 Pa), illumination (15–20 Lux), and a 12 h light/dark cycle, with three mice per cage. All experimental protocols were approved by the Animal Ethics Committee of China Agricultural University (Approval No.: AW51106202-5-02; Date: 15 January 2026) and conducted in accordance with the Guiding Principles for the Care and Use of Laboratory Animals. CYPs (purity ≥ 98%, catalog XY-XBZP-2608) were sourced from Shanghai Xuanya Bio-Technology Co., Ltd. (Shanghai, China) and prepared following previously established procedures [12].

### 2.2. Experimental Design and Animal Grouping

After adequate surgical training with negligible mortality, mice were randomly assigned to three groups (*n* = 10 per group): (1) sham-operated group (Sham), (2) myocardial I/R injury group (I/R), and (3) I/R+CYPs group (400 mg/kg). All animals were maintained on a standard chow diet throughout the experiment. Mice in the I/R+CYPs group received CYPs via daily intragastric administration (400 mg/kg) for 6 days, with the final dose given 60 min before induction of the myocardial I/R model. Mice in the Sham and I/R groups received an equal volume of ultrapure water via the same intragastric route on the same schedule. The primary outcome measure was myocardial infarct size assessed by means of TTC staining.

### 2.3. Myocardial I/R Injury Model

The myocardial MI/R injury model was established based on a previously described protocol with minor modifications [1]. All animal procedures complied with ARRIVE 2.0 and the 3Rs principle to minimize suffering. Preemptive subcutaneous buprenorphine and topical lidocaine gel were used for perioperative analgesia. Briefly, mice were fixed on a 37 °C warm platform under continuous 3% isoflurane inhalation anesthesia (1 L/min). After chest disinfection, left thoracotomy was performed between the 3rd and 4th intercostals, and the pericardium was gently opened to expose the LAD artery, which was ligated 1 mm below the left atrial appendage with 7-0 silk suture. Myocardial pallor confirmed successful ischemia. After 30 min ischemia, the suture was released for reperfusion, and all mice were sacrificed at 24 h post-reperfusion as the predefined experimental endpoint. Postoperative observation was conducted every 2 h within the first 12 h. Humane endpoints (>20% weight loss, severe dyspnea, refractory pain) were set; any affected mouse would be euthanized ahead of schedule. Refinement measures including gentle dissection, constant thermal support, and multimodal analgesia were adopted to reduce surgical trauma. Outcome assessment and data analysis were performed in a blinded manner.

### 2.4. Echocardiography and Hemodynamics

Cardiac structure and function were evaluated with a high-resolution in vivo micro-ultrasound system (Vevo 3100, FUJIFILM VisualSonics, Toronto, ON, Canada). Following anesthesia induction with 1.5% isoflurane, mice were positioned supine on a temperature-controlled platform. Heart rate was stabilized, when feasible, within a range of 450–500 bpm. Parasternal long-axis and short-axis views of the left ventricle (LV) were acquired at an imaging rate of 233 Hz. From end-systolic and end-diastolic LV dimensions, several functional indices were derived, including LV ejection fraction (LVEF), LV fractional shortening (LVFS), LV internal dimension at systole (LVIDs), LV end-systolic volume (LVESV), and cardiac output (CO). All imaging procedures and subsequent analyses were conducted by researchers who were blinded to group assignment.

### 2.5. Serum Biochemical Analysis

Whole blood samples were collected into 2 mL tubes containing clot activator (X0021, Xinkang Medical Equipment Co., Ltd., Taizhou, China) and allowed to clot at room temperature for 2 h. Subsequently, samples were centrifuged at 3000 rpm for 15 min at 2–8 °C, and the supernatant was harvested as serum. Serum levels of aspartate aminotransferase (AST), creatine kinase (CK), lactate dehydrogenase (LDH), and reactive oxygen species (ROS) were measured using commercial assay kits (AST: S03040, CK: S03024, LDH: S03034, Rayto, Shenzhen, China; ROS: G4306-48T, Servicebio). Lactate dehydrogenase isoenzyme 1 (LDH-1) and creatine kinase-MB (CK-MB) were determined using separate kits (C058-E and C060, respectively; Changchun Huili, Changchun, China).

### 2.6. Biochemical Assays

Serum and heart tissue samples were assessed for oxidative stress by measuring malondialdehyde (MDA) content, as well as the activities of superoxide dismutase (SOD) and glutathione peroxidase (GSH-Px). All assays were performed using commercial kits (MDA: Cat. No. A003-1-2; SOD: Cat. No. A001-3-2; GSH-Px: Cat. No. A005-1-2; Nanjing Jiancheng Technology, Nanjing, China) strictly following the manufacturer’s protocols. Absorbance values were recorded at 532 nm for MDA, 450 nm for SOD, and 412 nm for GSH-Px using a microplate reader (Thermo Fisher Scientific, Waltham, MA, USA).

### 2.7. TTC Staining

To delineate the area at risk, the coronary arteries were retrogradely perfused with 1.5% Evans blue dye. The heart was then subjected to freezing at −80 °C for 20 min. Following this, the heart was sliced transversely into 1 mm thick sections and incubated in 1% triphenyltetrazolium chloride (TTC) solution to visualize the infarct area. The staining reaction was terminated by fixation in 4% paraformaldehyde.

### 2.8. Histological Examination

Heart tissue samples were embedded in paraffin and sectioned at a thickness of 4 µm. The sections were subsequently deparaffinized, rehydrated, and subjected to hematoxylin and eosin (H&E) staining using a commercial kit (Servicebio, Wuhan, China) in accordance with the manufacturer’s protocols.

### 2.9. Transmission Electron Microscopy (TEM)

Freshly harvested heart tissues were immersed immediately in a fixative solution containing 2% glutaraldehyde and 2.5% paraformaldehyde in 0.1 M sodium cacodylate buffer (pH 7.4). Samples were fixed for 1 h at room temperature, followed by overnight incubation at 4 °C. After fixation, tissues were dehydrated through a graded ethanol series (30%, 50%, 70%, 90%, and 100%) and subsequently embedded in epoxy resin (Epon 812). Resin infiltration was performed under vacuum to remove air bubbles, and polymerization was carried out at 60 °C for 24–48 h to obtain solid blocks. Ultrathin sections (80–90 nm thickness) were cut using a Leica Ultracut E ultramicrotome (Leica Microsystems, Wetzlar, Germany), contrasted with 2% uranyl acetate and lead citrate (Sigma-Aldrich, St. Louis, MO, USA), and examined under a JEOL 1400 transmission electron microscope (Japan Electron Optics Laboratory Co., Ltd., Peabody, MA, USA) equipped with a Gatan Orius SC1000 digital CCD camera (Gatan, Pleasanton, CA, USA). Image analysis was performed using ImageJ software (v1.54f, NIH, Bethesda, MD, USA). For quantitative analysis of mitochondrial ultrastructure in transmission electron microscopy images, images were collected from the left ventricular ischemic border zone. For each mouse, five randomly selected non-overlapping fields were analyzed. Mitochondrial ultrastructural alterations, including mitochondrial swelling, cristae disruption, vacuolization, and membrane damage, were assessed. All analyses were performed by investigators blinded to the experimental groups.

### 2.10. RNA Extraction and Real-Time Quantitative PCR (RT-qPCR)

Total RNA was extracted from mouse heart tissues using TRIzol reagent (Thermo Fisher Scientific, Waltham, MA, USA) according to the manufacturer’s instructions. RNA purity and concentration were assessed by measuring the absorbance ratio at 260/280 nm using a Nanodrop spectrophotometer (Thermo Fisher Scientific, Waltham, MA, USA). For each sample, 1 μg of total RNA was reverse-transcribed into complementary DNA (cDNA) using HiScript III RT SuperMix (R323-01, Vazyme, Nanjing, China) or Reverse Transcription Reagent kits (FT301, Vazyme) following the manufacturer’s protocols.

Real-time quantitative PCR (RT-qPCR) was performed using SYBR Green Master Mix (Q711-02 or Q712-02, Vazyme, Nanjing, China) on an Applied Biosystems StepOnePlus Real-Time PCR System (ABI 7500, Thermo Fisher Scientific). The thermal cycling conditions were set as follows: initial denaturation at 95 °C for 5 min, followed by 40 cycles of 95 °C for 10 s and 60 °C for 30 s. All reactions were carried out in triplicate. Prior to formal experiments, primer specificity was validated by means of melting curve analysis, and amplification efficiency was evaluated to ensure optimal reaction conditions. Relative mRNA expression levels were calculated using the comparative cycle threshold (2^−ΔΔCt^) method, with β-actin (Actb) serving as the internal reference gene. Primer sequences for target genes were synthesized by Beijing Genomics Institution (Beijing, China) and are listed in Supplementary Table S1.

### 2.11. ATP Quantification

ATP levels in cardiac tissues and cardiomyocytes were measured using an Enhanced ATP Assay Kit (S0027, Beyotime, Shanghai, China) according to the manufacturer’s instructions. Briefly, tissue samples and cells were lysed in ATP detection lysis buffer, followed by centrifugation at 12,000 rpm for 5 min at 4 °C. The resulting supernatant was collected for ATP quantification. Luminescence intensity was measured using a microplate reader (BioTek Instruments, Winooski, VT, USA), and ATP concentrations were normalized to total protein content determined using a BCA Protein Assay Kit (CW0014S, CWBIO, Beijing, China).

### 2.12. Gut Microbiota Analysis

Colonic contents were collected for bacterial genomic DNA extraction, 16S rRNA gene amplification, PCR product validation, and library sequencing, all performed according to standard protocols at Majorbio Laboratory (Shanghai, China). The V3–V4 hypervariable regions of the 16S rRNA gene were amplified using the primer pair 338F (ACTCCTACGGGAGGCAGCAG) and 806R (GGACTACHVGGGTWTCTAAT). Sequencing data were analyzed on the Majorbio Cloud Platform (https://cloud.majorbio.com). High-quality reads were clustered into operational taxonomic units (OTUs), and downstream analyses including alpha and beta diversity metrics were conducted using the QIIME software package (version 1.2.8).

Linear discriminant analysis effect size (LEfSe) was employed to identify differentially abundant microbial taxa across all classification levels between the saline and I/R+CYPs groups. Alpha diversity indices (ACE and Shannon) and beta diversity based on principal coordinates analysis (PCoA) were calculated to assess community richness and structure. PCoA was performed using the Adonis statistical method. Microbial composition and relative abundance were characterized at both the phylum and genus levels. Spearman correlation heatmap analysis was conducted to evaluate associations between gut microbiota and serum lipid profiles as well as myocardial I/R injury pathological parameters. Functional pathways of the microbial communities were predicted using PICRUSt (Phylogenetic Investigation of Communities by Reconstruction of Unobserved States) based on comparisons with the Kyoto Encyclopedia of Genes and Genomes (KEGG) database.

### 2.13. Statistical Analysis

Data are expressed as mean ± standard deviation (SD). Data were analyzed under the assumption of a normal Gaussian distribution, and homogeneity of variance was assessed before statistical analysis using the Brown–Forsythe test and Bartlett’s test. Statistical comparisons among the Sham, I/R, and I/R+CYPs groups were performed using one-way analysis of variance (ANOVA), followed by Tukey’s multiple comparisons test for pairwise comparisons among groups. Adjusted *p* values were used to account for multiple comparisons, and 95% confidence intervals (CIs) of the mean differences were calculated where appropriate. The coefficient of determination (R^2^) from ANOVA was also reported to indicate the proportion of variance explained by group differences. All analyses were conducted with GraphPad Prism software (version 10.0, GraphPad Software, San Diego, CA, USA). A value of *p* < 0.05 was considered statistically significant.

## 3. Results

### 3.1. CYPs Pretreatment Improves Cardiac Function After Myocardial I/R Injury

**Figure 1:**
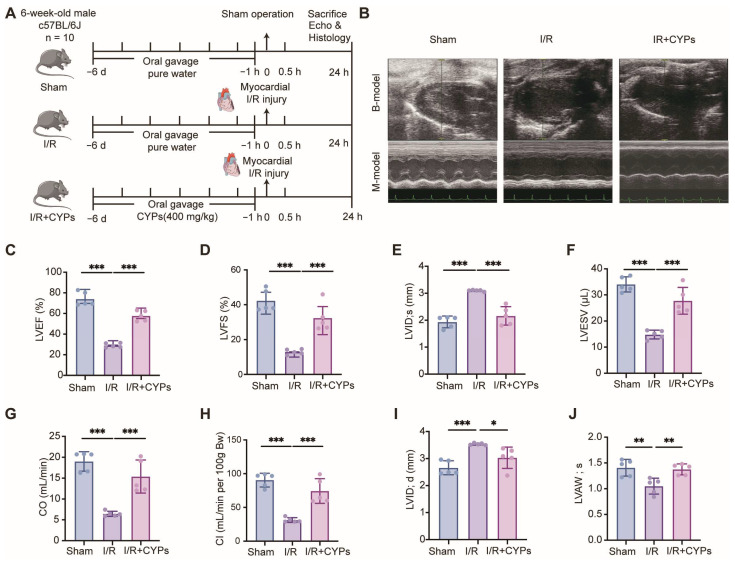
Experimental design and representative results of CYPs treatment on myocardial I/R injury in mice. (**A**) Schematic diagram of experimental design and grouping. Six-week-old male C57BL/6J mice were randomly divided into Sham, I/R, and I/R+CYPs groups (*n* = 10 per group). I/R+CYPs group was administered 400 mg/kg CYPs by gavage 6 days and 1 h before ischemia; Sham and I/R groups received vehicle. All mice underwent 30 min myocardial ischemia followed by reperfusion and were sacrificed 24 h later for analysis. (**B**) Representative B-mode and M-mode echocardiographic images. (**C**–**J**) Quantitative analyses of left ventricular ejection fraction (LVEF), fractional shortening (LVFS), end-systolic internal diameter (LVID; s), end-systolic volume (LVESV), cardiac output (CO), cardiac index (CI), end-diastolic internal diameter (LVID; d), and systolic anterior wall thickness (LVAW; s) (*n* = 5). Data are mean ± SD; one-way ANOVA with post hoc tests, *** *p* < 0.001, ** *p* < 0.01, * *p* < 0.05.

To evaluate the protective effect of CYPs against myocardial I/R injury, we established a mouse model of myocardial I/R and administered CYPs (400 mg/kg) by gavage once daily for 6 days, with an additional dose given 1 h before ischemia (Figure 1A). Echocardiography was performed 24 h after surgery. Representative B-mode and M-mode images showed that, compared with the Sham group, mice in the I/R group exhibited marked enlargement of the cardiac chambers and reduced ventricular wall motion. In the CYP-treated group, cardiac chamber enlargement was attenuated and ventricular wall motion was improved (Figure 1B).

Quantitative analysis showed that left ventricular ejection fraction (LVEF) and left ventricular fractional shortening (LVFS) were lower in the I/R group than in the Sham group (* *p* < 0.001), indicating that I/R injury caused severe cardiac dysfunction. CYPs pretreatment enhanced these parameters, with both LVEF and LVFS markedly higher than those in the I/R group (*p* < 0.01; Figure 1C,D). In addition, left ventricular end-systolic diameter (LVID; s) and left ventricular end-systolic volume (LVESV) were elevated in the I/R group, whereas CYPs treatment effectively attenuated cardiac chamber dilation (Figure 1E,F). Cardiac output (CO) and cardiac index (CI) were reduced in the I/R group but were markedly restored following CYPs intervention (Figure 1G,H). No significant differences were observed among the groups in left ventricular end-diastolic diameter (LVID; d) or left ventricular anterior wall thickness at systole (LVAW; s) (Figure 1I,J). Taken together, these findings suggest that CYPs pretreatment was associated with improved cardiac function after myocardial I/R injury, indicating a potential protective effect against acute myocardial damage.

### 3.2. CYPs Attenuate Myocardial I/R Injury

**Figure 2:**
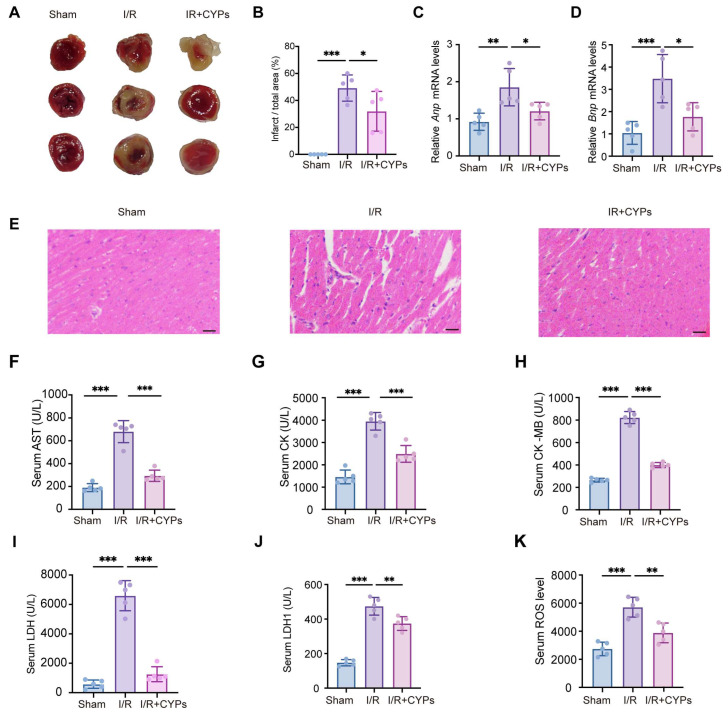
CYPs alleviate myocardial I/R injury. Representative triphenyltetrazolium chloride (TTC)-stained myocardial sections (**A**) and quantitative analysis of infarct area as a percentage of total myocardial area (**B**) from Sham, I/R, and I/R+CYPs mice are shown. Viable tissue is stained red, while infarcted tissue appears pale in TTC staining. Relative mRNA expression levels of *Anp* (**C**) and *Bnp* (**D**) in the heart (*n* = 5). (**E**) Representative H&E-stained myocardial sections (scale bar: 20 μm). (**F**–**J**) Serum levels of AST (**F**), CK (**G**), CK-MB (**H**), LDH (**I**), and LDH1 (**J**) (*n* = 5). (**K**) Serum ROS levels (*n* = 5). Data are presented as mean ± SD, with statistical significance determined via one-way ANOVA followed by post hoc tests (*** *p* < 0.001, ** *p* < 0.01, * *p* < 0.05).

To evaluate the cardioprotective effects of CYPs against myocardial I/R injury, we assessed myocardial infarct size, cardiac injury markers, and oxidative stress levels. As shown in Figure 2A,B, infarct size was larger in the I/R group than in the Sham group (*p* < 0.001), whereas CYPs pretreatment markedly reduced infarct size (*p* < 0.01). Consistent with these findings, the mRNA expression levels of hypertrophic markers, including atrial natriuretic peptide (*Anp*, Figure 2C) and brain natriuretic peptide (*Bnp*, Figure 2D), were upregulated in the I/R group (*p* < 0.001), and this increase was attenuated by CYPs treatment (*p* < 0.05). Hematoxylin and eosin (H&E) staining revealed marked myocardial structural disruption and inflammatory cell infiltration in the I/R group, both of which were ameliorated in the I/R+CYPs group (Figure 2E). We further measured serum cardiac enzyme levels as indicators of myocardial injury. Compared with the Sham group, the I/R group exhibited elevated levels of aspartate aminotransferase (AST, Figure 2F), creatine kinase (CK, Figure 2G), creatine kinase-MB (CK-MB, Figure 2H), lactate dehydrogenase (LDH, Figure 2I), and lactate de-hydrogenase 1 (LDH1, Figure 2J) (*p* < 0.001). CYPs administration lowered these elevated enzyme levels (*p* < 0.05) or (*p* < 0.01). In addition, serum reactive oxygen species (ROS) levels were markedly elevated in the I/R group relative to the Sham group (*p* < 0.001), and this increase was significantly suppressed by CYPs treatment (*p* < 0.05; Figure 2K). Collectively, these results suggest that CYPs pretreatment is associated with cardioprotective effects against myocardial I/R injury, accompanied by reduced oxidative stress and myocardial damage.

### 3.3. CYPs Attenuate Oxidative Stress in Myocardial I/R Injury

**Figure 3:**
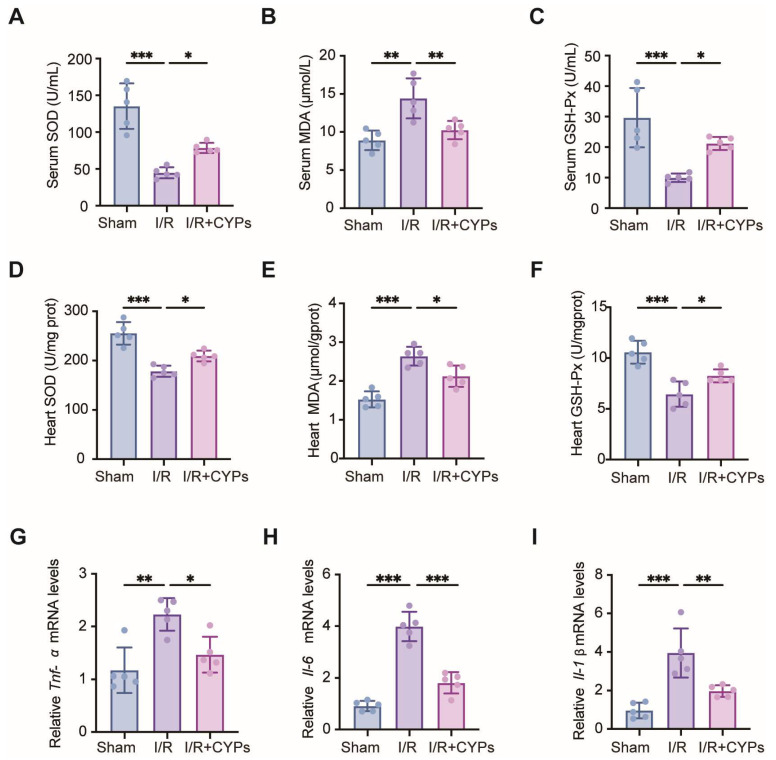
CYPs improve oxidative stress levels and attenuate inflammatory factors in mice with myocardial I/R injury. (**A**–**C**) Serum levels of SOD (**A**), MDA (**B**), and GSH-Px (**C**) (*n* = 5); (**D**–**F**) heart tissue levels of SOD (**D**), MDA (**E**), and GSH-Px (**F**) (*n* = 5). (**G**–**I**) Relative mRNA expression levels of *Tnf-α* (**G**), *Il-6* (**H**), and *Il-1β*(**I**) in cardiac tissue (*n* = 5). All data are presented as mean ± SD. Statistical analysis was performed with one-way ANOVA. *** *p* < 0.001, ** *p* < 0.01, * *p* < 0.05.

Previous studies have demonstrated that CYPs possess potent antioxidant properties. To evaluate whether CYPs treatment is associated with reduced oxidative stress-related changes following myocardial I/R injury, we measured the activities of antioxidant enzymes and the levels of oxidative stress markers in both serum and cardiac tissue from I/R-injured mice. Compared with the Sham group, mice in the I/R group exhibited significantly increased malondialdehyde (MDA) levels, accompanied by markedly decreased activities of superoxide dismutase (SOD) and glutathione peroxidase (GSH-Px) in both serum and myocardial tissue. However, CYPs pretreatment significantly increased SOD and GSH-Px activities while reducing MDA levels in both serum and cardiac tissue from I/R-injured mice, with all changes showing statistically significant differences compared with the I/R group (*p* < 0.001) (Figure 3A–F). These results suggest that CYPs pretreatment is associated with attenuation of oxidative stress induced by myocardial I/R injury. We next investigated whether CYPs were associated with changes in the inflammatory response following myocardial I/R injury. As shown in Figure 3G–I, the mRNA expression levels of pro-inflammatory cytokines, including tumor necrosis factor-α (*Tnf-α*), interleukin-6 (*Il-6*), and interleukin-1β (*Il-1β*), were significantly upregulated in the cardiac tissue of mice in the I/R group compared with the Sham group (*p* < 0.001). However, CYPs pretreatment markedly suppressed the expression of these inflammatory mediators, with *Tnf-α*, *Il-6*, and *Il-1β* mRNA levels all significantly lower in the I/R+CYPs group than in the I/R group (*p* < 0.001; Figure 3G–I). These results suggest that CYPs pretreatment may attenuate I/R-associated cardiac inflammation, as reflected by decreased pro-inflammatory cytokine expression.

### 3.4. CYPs Preserve Mitochondrial Function to Attenuate Myocardial I/R Injury

**Figure 4:**
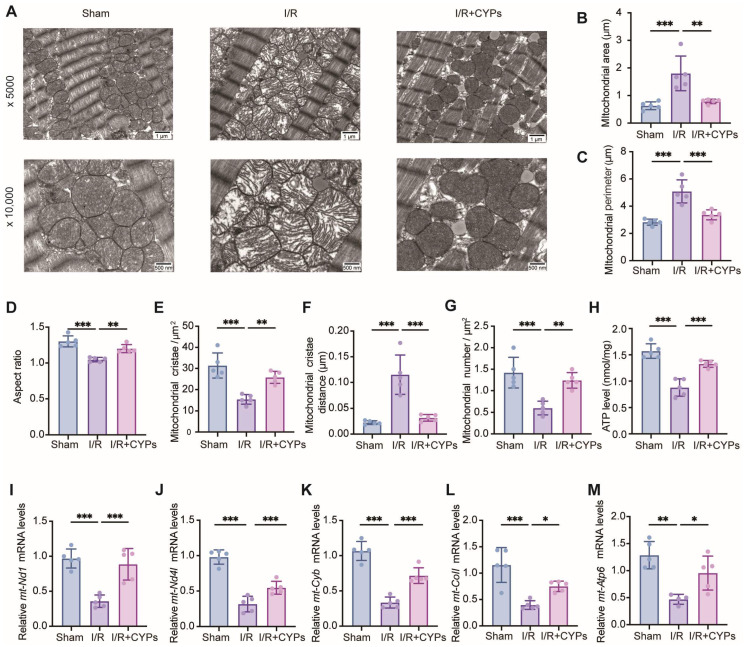
CYPs ameliorate mitochondrial structure and function in mice with myocardial I/R injury. (**A**) Representative transmission electron microscopy (TEM) images of myocardial mitochondria (scale bars: 5000× and 10,000× magnification). Quantitative analyses of mitochondrial morphological parameters include (**B**) mitochondrial area (μm^2^), (**C**) mitochondrial perimeter (μm), (**D**) aspect ratio, (**E**) mitochondrial cristae number per μm^2^, (**F**) mitochondrial cristae distance (μm), (**G**) mitochondrial number per μm^2^, and (**H**) ATP level (nmol/mg) (*n* = 5). (**I**–**M**) Relative mRNA expression levels of mitochondrial genes *mt-Nd1* (**I**), *mt-Nd4L* (**J**), *mt-Cyb* (**K**), *mt-CoII* (**L**), and *mt-Atp6* (**M**) in cardiac tissue (*n* = 5). All data are presented as mean ± SD. Statistical analysis was performed with one-way ANOVA followed by posthoc tests. *** *p* < 0.001, ** *p* < 0.01, * *p* < 0.05.

To further explore mitochondrial changes associated with the cardioprotective effects of CYPs, we examined mitochondrial morphology and function in myocardial tissue following I/R injury. Transmission electron microscopy revealed severe mitochondrial damage in the I/R group, characterized by swelling, cristae disorganization, and membrane disruption, whereas CYPs pretreatment was associated with improved mitochondrial ultrastructure (Figure 4A). Quantitative analysis confirmed these observations. Compared with the Sham group, mitochondria in the I/R group exhibited enlarged area and perimeter, along with a reduced aspect ratio, indicating swelling (*p* < 0.001). CYPs treatment significantly attenuated these abnormalities (*p* < 0.01 or *p* < 0.001; Figure 4B–D). Additionally, mitochondrial cristae number was decreased and cristae distance was increased in the I/R group (both *p* < 0.001), indicating disorganization, and these alterations were partially attenuated by CYPs pretreatment (*p* < 0.01 or *p* < 0.001; Figure 4E,F). Mitochondrial number was also reduced in the I/R group (*p* < 0.001), and this reduction was ameliorated by CYPs treatment (*p* < 0.01; Figure 4G).

Consistent with the morphological changes, myocardial ATP levels were lower in the I/R group than in the Sham group (*p* < 0.001), indicating mitochondrial dysfunction. CYPs pretreatment increased ATP levels compared with the I/R group (*p* < 0.01; Figure 4H). We further examined the mRNA expression of mitochondrial-encoded genes. As shown in Figure 4I–M, the expression levels of *mt-Nd1*, *mt-Nd4L*, *mt-Cyb*, *mt-CoII*, and *mt-Atp6* were all downregulated in the I/R group compared with the Sham group (*p* < 0.001), while CYPs treatment enhanced their expression (*p* < 0.01 or *p* < 0.001). Collectively, these results suggest that CYPs pretreatment is associated with preservation of mitochondrial morphology, improved ATP levels, and upregulated mitochondrial gene expression, which may be involved in its cardioprotective effects against myocardial I/R injury.

### 3.5. CYPs Regulate the Gut Microbiota

**Figure 5:**
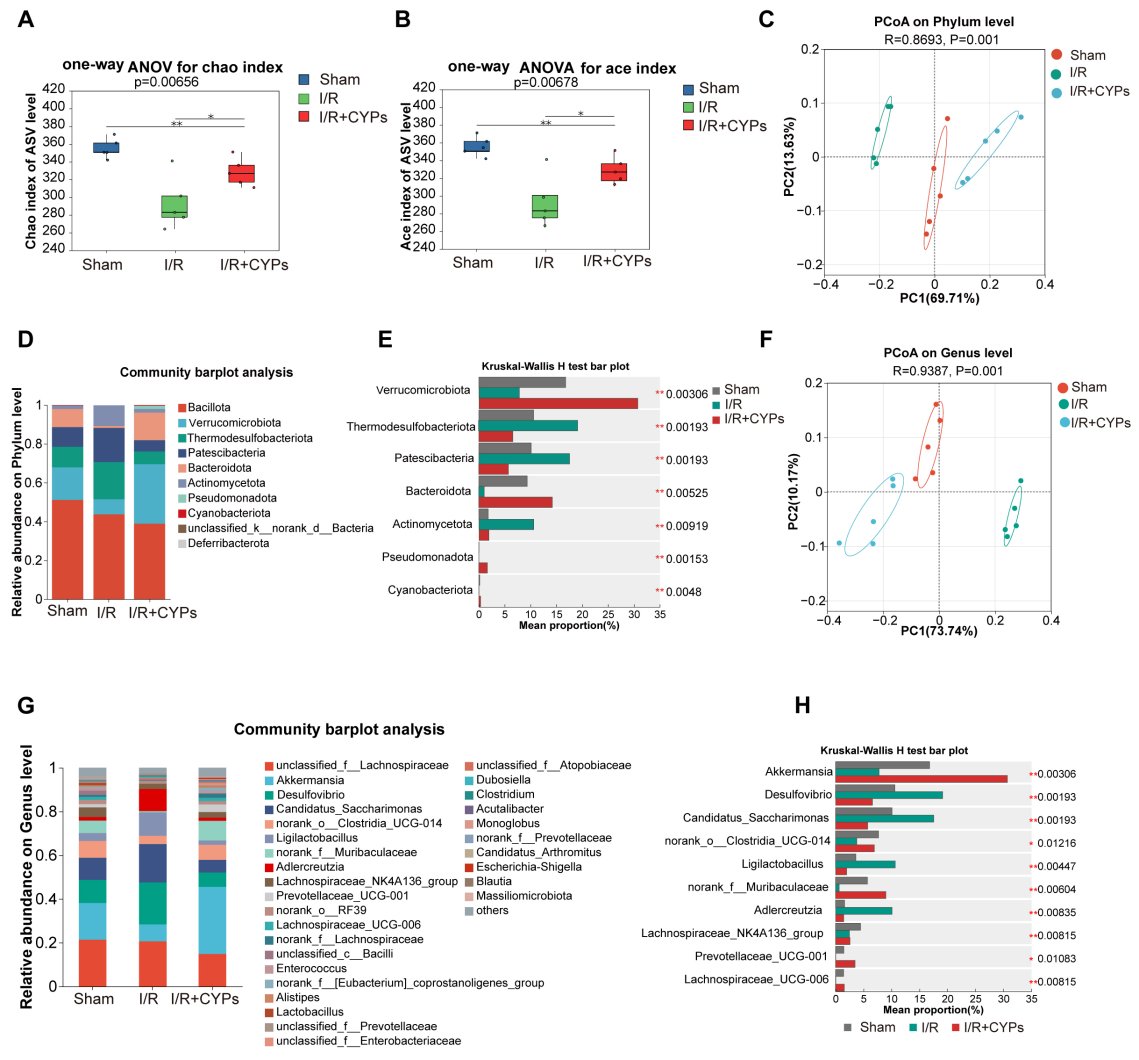
CYPs remodel gut microbial community structure in mice with myocardial I/R injury. (**A**,**B**) Gut microbial alpha diversity, including (**A**) Chao index and (**B**) Ace index (*n* = 5). (**C**–**E**) Gut microbial community analysis at the phylum level: (**C**) PCoA (R = 0.8693, *p* = 0.001); (**D**) Relative abundance of gut microbiota at the phylum level; (**E**) Kruskal–Wallis H test analysis of differentially abundant phyla. (**F**–**H**) Gut microbial community analysis at the genus level: (**F**) PCoA (R = 0.9387, *p* = 0.001); (**G**) Relative abundance of gut microbiota at the genus level; (**H**) Kruskal—Wallis H test analysis of differentially abundant genera. All data are presented as mean ± SEM. Statistical analysis was performed with one-way ANOVA followed by post hoc tests or the Kruskal–Wallis H test. ** *p* < 0.01, * *p* < 0.05.

Compared to the Sham group, the I/R group showed significantly reduced Chao and Ace indices, indicating a decrease in gut microbial richness (Figure 5A,B). CYPs treatment markedly restored both indices, suggesting that CYPs alleviated the loss of microbial richness induced by myocardial I/R injury (Figure 5A,B). PCoA analysis (R = 0.8693, *p* = 0.001; Figure 5C) revealed that CYPs treatment reversed the injury-induced microbial shift, clustering with the Sham group and distinct from the I/R group. The phylum-level bar plot (Figure 5D) further demonstrated that CYPs reshaped the dominant gut microbial composition. The Kruskal–Wallis H test (Figure 5E) identified key phyla that were significantly altered by injury and CYPs intervention. Injury increased *Thermodesulfobacteriota* in the I/R group, and this change was reversed by CYPs. In contrast, CYPs increased *Verrucomicrobiota* and *Bacteroidota* and decreased *Thermodesulfobacteriota*, *Patescibacteria*, and *Actinomycetota*. PCoA analysis (Figure 5F) based on Bray–Curtis distance revealed significant differences among the three groups (PERMANOVA: R = 0.9387, *p* = 0.001). The genus-level composition bar plot (Figure 5G) further revealed shifts in dominant taxa following CYPs treatment. The Kruskal–Wallis H test (Figure 5H) identified genera significantly modulated by CYPs (*p* < 0.05 or *p* < 0.01). Notably, CYPs reduced the abundance of *Desulfovibrio*, which was enriched in the I/R group and is associated with intestinal inflammation. In contrast, CYPs increased beneficial genera, including *Akkermansia*, *Muribaculaceae*, and *Lachnospiraceae*, all of which were decreased in the I/R group. Given the limited sample size used for microbiota analysis, these findings should be interpreted as exploratory evidence of CYP-associated microbial alterations. These results indicate that CYPs remodel the gut microbiome, promoting a favorable microbial profile that may confer protection against myocardial I/R injury.

### 3.6. CYPs Affect the Functions of Gut Microbiota

**Figure 6:**
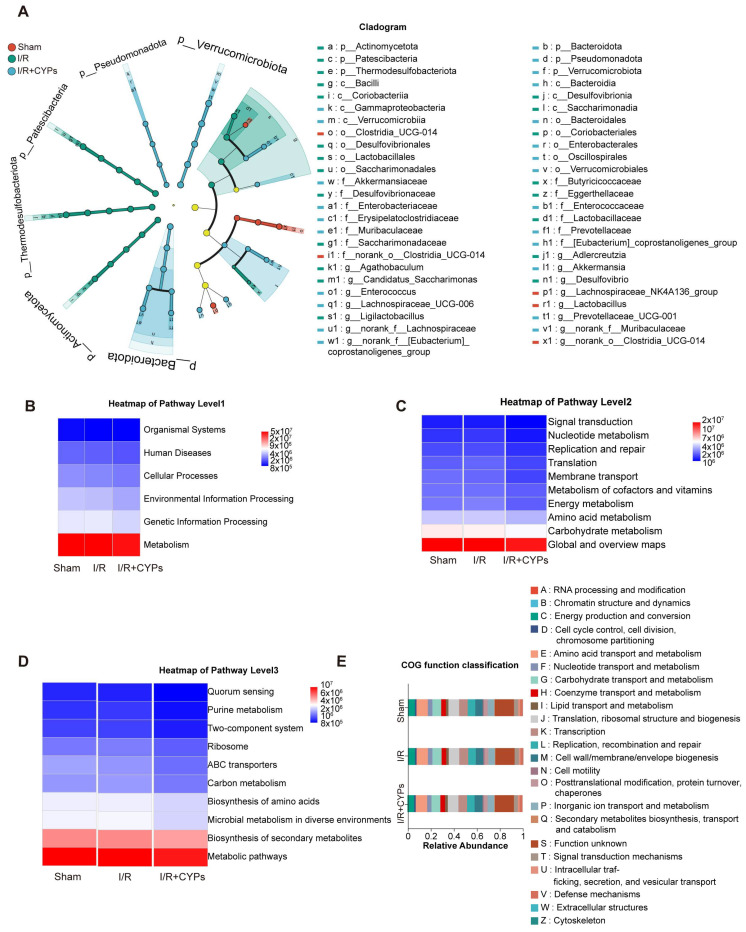
CYPs remodel gut microbial composition and identify key differential taxa in mice with myocardial I/R injury. (**A**) Cladogram illustrating the phylogenetic distribution of gut microbial taxa with differential abundance across Sham, I/R, and I/R+CYPs groups. Nodes and dots are colored to indicate taxa significantly enriched in each group (red: Sham, blue: I/R, green: I/R+CYPs), with hierarchical levels ranging from phylum (inner circles) to genus (outer circles). Functional prediction of gut microbial communities based on KEGG pathways: (**B**) Functional pathway analysis at Level 1; (**C**) functional pathway analysis at Level 2; (**D**) functional pathway analysis at Level 3. The color intensity indicates the relative abundance of each pathway (red: high abundance, blue: low abundance). (**E**) COG functional classification of gut microbial community. The horizontal axis indicates the relative abundance of each COG functional category, and the vertical axis lists the COG functional categories (A–Z) with corresponding biological functions as shown.

To further identify the key microbial lineages modulated by CYPs, a cladogram-based taxonomic analysis was performed. Compared with the I/R group, CYPs enriched multiple beneficial microbial lineages, including *Verrucomicrobiota* and *Bacteroidota*, while suppressing injury-associated taxa such as *Thermodesulfobacteriota*, *Patescibacteria*, and *Actinomycetota*. These lineage-specific alterations suggest that CYPs may reshape the potential functional profile of the gut microbiota, prompting us to perform PICRUSt-based functional prediction analysis (Figure 6A).

To further explore the predicted functional profile effect of CYPs on the function of gut microbiota, KEGG pathway analysis via PICRUSt was performed to infer the potential functions of bacterial communities, and the association between differential bacteria and predicted functional pathways was evaluated. KEGG analyses at pathway levels 1, 2, and 3 revealed that the most abundant pathways were Metabolism, Global and overview maps, and Metabolic pathways, respectively (Figure 6B–D), with no obvious differences in the overall predicted functional profile observed among the Sham, I/R, and I/R+CYPs groups. COG functional annotation analysis revealed that the gut microbial community was predicted to contain various COG functional categories (A–Z) spanning multiple biological processes, with the relative abundances of each functional group shown in Figure 6E.

### 3.7. Correlation Analysis Between Gut Microbiota and Cardiac Metabolic Phenotypes

**Figure 7:**
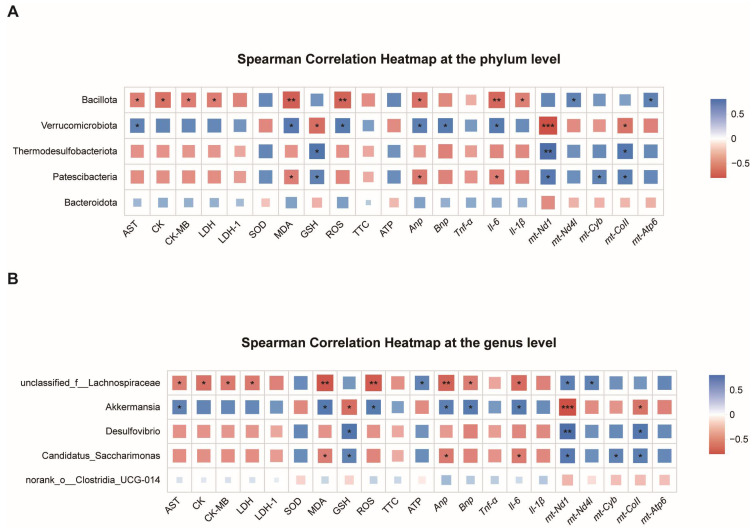
Spearman correlation analysis of gut microbiota with myocardial I/R markers in a mouse model of myocardial I/R injury. Spearman correlation heatmaps between gut microbiota and myocardial injury-related indicators in mice with myocardial I/R injury. (**A**) Correlation analysis at the phylum level; (**B**) correlation analysis at the genus level. The color gradient represents the Spearman correlation coefficient, with asterisks indicating statistical significance (* *p* < 0.05, ** *p* < 0.01, *** *p* < 0.001).

To explore the potential links between gut microbiota and cardiac functional parameters, we performed Spearman correlation analysis between key microbial taxa (at both phylum and genus levels) and cardiac injury markers, oxidative stress indicators, inflammatory cytokines, and mitochondrial function-related genes (Figure 7A,B). Spearman correlation analysis between gut microbiota at the phylum level and biochemical indicators showed that *Bacillota* was negatively associated with AST, CK, CK-MB, LDH, MDA, ROS, *Anp*, *Il-6*, and *Il-1β* and positively correlated with *mt-Nd4l* and *mt-Atp6*. *Thermodesulfobacteriota* showed positive correlations with AST, MDA, ROS, *Anp*, *Bnp*, and *Il-6* and negative correlations with GSH-Px, *mt-Nd1*, and *mt-CoII*; *Verrucomicrobiota* was positively correlated with GSH-Px, *mt-Nd1*, and *mt-CoII*; *Patescibacteria* was significantly negatively correlated with MDA, *Anp*, and *Il-6* and positively correlated with GSH-Px, *mt-Nd1*, *mt-Cyb*, and *mt-CoII*. Distinct association patterns were observed between different bacterial phyla and host indicators. Spearman correlation analysis between gut microbiota at the genus level and biochemical indicators revealed that *unclassified_f__Lachnospiraceae* was negatively associated with AST, CK, CK-MB, LDH, MDA, ROS, *Anp*, *Bnp*, and *Il-6* and positively correlated with ATP, *mt-Nd1*, and *mt-Nd4l. Desulfovibrio* was positively correlated with AST, MDA, ROS, *Anp*, *Bnp*, and *Il-6*, and negatively correlated with GSH-Px, *mt-Nd1*, and *mt-CoII*. *Akkermansia* was positively correlated with GSH-Px, *mt-Nd1*, and *mt-CoII*. *Candidatus_Saccharimonas* was negatively correlated with MDA, *Anp*, and *IL-6* and positively correlated with GSH-Px, *mt-Nd1*, mt-*Cyb*, and *mt-CoII*. Distinct association patterns were observed between different bacterial genera and host indicators related to tissue injury, oxidative stress, inflammation, and mitochondrial function. Collectively, these correlation results suggest that CYP-associated alterations in gut microbiota composition are closely associated with reduced cardiac injury, alleviated oxidative stress and inflammation, and improved mitochondrial function, indicating a potential link between gut microbiota remodeling and the cardioprotective effects of CYPs against myocardial I/R injury.

## 4. Discussion

Myocardial I/R injury remains a major clinical challenge. Although reperfusion therapies, including thrombolysis and percutaneous coronary intervention, are essential for restoring blood flow after ischemic events, they may paradoxically aggravate myocardial damage, and effective adjunctive strategies to limit reperfusion-induced injury remain limited [13]. Therefore, identifying interventions capable of attenuating myocardial I/R injury is of considerable clinical relevance. In the present study, CYPs exerted a marked cardioprotective effect against myocardial I/R injury, accompanied by reduced oxidative stress and inflammatory responses, as well as preservation of mitochondrial ultrastructure and respiratory function. The CYPs used in the present study were consistent with those described in our previous reports [12]. Their biological activity is generally considered to be associated with molecular weight, monosaccharide composition, and functional group characteristics [14]. Characterization analyses showed that the CYPs used herein had an average molecular weight of 137.15 kDa and previously demonstrated lipid-lowering and anti-inflammatory activities, broadly resembling the bioactive properties reported for Pseudostellaria heterophylla polysaccharides within a molecular weight range of 50–210 kDa [15,16]. Previous structural studies further suggest that the antioxidant activity of CYPs may be related to their relatively high glucuronic acid content and abundant hydroxyl groups [17], while methylation has been reported to enhance antioxidant activity and modify physicochemical properties. However, the specific structural determinants underlying the cardioprotective effects observed in the present study remain to be further defined.

The dose used in this study was selected on the basis of previous findings showing that CYPs at 100, 200, and 400 mg/kg dose-dependently alleviated colitis, with the most pronounced efficacy observed at 400 mg/kg [18]. Accordingly, 400 mg/kg was adopted in the present study as an empirically supported effective dose. However, because dose–response relationships were not evaluated in the myocardial I/R model, the optimal dose for cardioprotection remains to be established. Only male mice were included in this study to reduce potential variability associated with sex-related differences in inflammatory and oxidative stress responses [19]. In future studies, female mice will also be taken into consideration to further evaluate the potential impact of sex-dependent differences on the observed effects. A positive control was not included in this study. Therefore, although the present findings support the cardioprotective potential of CYPs, direct comparison with currently available cardioprotective agents was not possible and should be addressed in future studies. Given that the primary objective of this study was to evaluate the protective effects of the test agent, the absence of a positive control does not affect the interpretation of the efficacy findings [18]. Therefore, the present study captures only the acute-stage effects of CYPs. Future studies incorporating multiple reperfusion time points, including earlier time points to assess the initiation of oxidative and inflammatory injury and later time points to evaluate sustained mitochondrial protection, gut microbiota remodeling, metabolite changes, and post-I/R cardiac remodeling, are needed to more comprehensively define the temporal profile and durability of CYP-mediated cardioprotection. From a translational standpoint, CYPs present favorable clinical prospects—their natural provenance and the absence of observable adverse effects in this study support their potential as a safe adjunctive intervention. Furthermore, as a plant with both medicinal and dietary applications, Chinese yam extracts have seen extensive use in functional food products [20,21]. The effects of long-term CYPs intervention on myocardial I/R injury remain unexplored, although cumulative protection is plausible based on its sustained bioactivity. Preventive efficacy does not directly translate to therapeutic efficacy, yet post-ischemic administration of CYPs may still confer benefits if given within an early time window, a hypothesis requiring experimental validation. Prospective clinical trials will be essential to substantiate these preclinical observations and refine therapeutic protocols.

The core pathological process of myocardial I/R injury involves the progressive amplification of oxidative stress, inflammatory responses, and mitochondrial dysfunction, ultimately leading to cardiomyocyte death and pathological cardiac remodeling [22,23,24]. Owing to the relatively low activity of antioxidant enzymes, cardiac tissue is particularly vulnerable to oxidative stress. Antioxidant enzymes and free radical scavengers have been shown to alleviate cardiac dysfunction, reduce infarct size, and limit the progression of myocardial infarction [25]. Elevated myocardial Anp and Bnp levels indicate localized ventricular wall stress and cardiomyocyte injury severity following I/R, representing both a marker of damage extent and an activated endogenous cardioprotective response [26]. With respect to serum biomarkers of myocardial injury, CYPs intervention significantly attenuated multiple indices associated with cardiomyocyte membrane integrity, including CK, CK-MB, LDH, LDH-1, and AST, concomitant with a marked reduction in circulating ROS levels [27]. Collectively, these findings suggest that CYPs pretreatment is associated with reduced oxidative injury and membrane disruption in cardiomyocytes, and this association may contribute to structural preservation and functional improvement, as reflected by increased LVEF and LVFS.

In the pathological progression of myocardial I/R injury, oxidative stress and inflammatory responses are closely intertwined and together constitute a central driving force in myocardial damage [28]. The results of the present study indicate that CYPs intervention significantly reversed the oxidative imbalance induced by I/R, restored the activities of SOD and GSH-Px in both myocardial tissue and serum, and simultaneously reduced MDA levels. Consistent with previous reports showing that polysaccharide compounds exert antioxidant effects through free radical scavenging, the antioxidant action of CYPs was not confined to the local myocardium but also extended to the systemic circulation. This feature is of considerable clinical relevance [29]. Furthermore, the suppressive effect of CYPs on inflammatory responses further supports its multitarget mode of action; the mRNA expression levels of Tnf-α, Il-6, and Il-1β in myocardial tissue were significantly downregulated, suggesting that CYPs may exert a synergistic cardioprotective effect by concurrently interrupting oxidative stress and inflammatory cascade activation [30].

Given that oxidative stress initiates and propagates mitochondrial damage in myocardial I/R injury [31], we further examined CYP-mediated regulation of mitochondrial architecture and function, with pathological changes verified via transmission electron microscopy. Transmission electron microscopy revealed substantial mitochondrial structural damage in the I/R group, characterized by mitochondrial swelling, cristae disarray, and compromised membrane integrity. CYPs pretreatment attenuated these abnormalities and was associated with preserving mitochondrial count and normalizing morphological parameters including area and aspect ratio, alongside increased cristae density and reduced cristae spacing [32]. Mitochondrial structural stabilization may contribute to mitigating myocardial I/R injury. CYP-mediated architectural restoration preserves respiratory chain complex integrity and electron transport efficiency, thereby potentially supporting oxidative phosphorylation, attenuating ATP depletion and ROS burst, and ultimately meeting cardiomyocyte energy demands while suppressing cell death [33,34]. This structural restoration accounts for the upregulated expression of respiratory chain complex genes (*mt-Nd1*, *mt-Nd4l*, *mt-Cyb*, *mt-CoII*, *mt-Atp6*) by CYPs. The I/R-induced downregulation of these genes is consistent with ATP depletion [35]. Conversely, CYPs partially reversed oxidative phosphorylation dysfunction and restored cardiac ATP levels, suggesting that mitochondrial protection may be involved in the cardioprotective effects of CYPs [36]. However, because direct functional assays, such as respiratory chain complex activity, oxygen consumption rate measurement, or mitochondrial respiration analysis, were not performed, the present findings mainly support the preservation of mitochondrial integrity and bioenergetic status and provide only indirect evidence of improved mitochondrial function.

Emerging evidence has consistently linked daily dietary habits to the structural configuration of the intestinal microbiota [37]. The gut microbiota plays a significant role in the progression and expansion of I/R diseases [38]. In the present study, CYPs supplementation reshaped the gut microbiota at the genus level in mice subjected to myocardial I/R injury. Concurrently, CYPs also suppressed the I/R-induced enrichment of dysbiosis-associated genera, including *Ligilactobacillus* and *Adlercreutzia*, all of which were significantly represented in the I/R group and linked to gut inflammation and microbial dysbiosis [39,40,41,42,43]. Specifically, CYPs reduced the abundance of the pro-inflammatory genus *Desulfovibrio*, which was significantly enriched in the I/R model, while markedly increasing the relative abundance of key beneficial genera including *Akkermansia, norank_f_Muribaculaceae, Prevotellaceae_UCG-001, Lachnospiraceae_UCG-006*, and *Lachnospiraceae_NK4A136_group*. These taxa have been associated with gut dysbiosis, and their reduction following CYPs treatment may contribute to the restoration of microbial homeostasis [12,44,45,46,47]. In addition, CYPs enriched beneficial SCFA (Short-Chain Fatty Acid)-producing bacteria, which may be associated with promoting intestinal homeostasis and systemic anti-inflammatory effects, and also increased *Akkermansia*, a probiotic genus potentially associated with improved barrier integrity, reduced metabolic inflammation, and enhanced antioxidant capacity [48]. CYPs likewise suppressed the overgrowth of *Desulfovibrio*, a pro-inflammatory genus closely linked to dysbiosis. The coordinated enrichment of SCFA-producing bacteria and *Akkermansia*, together with the suppression of dysbiosis-associated taxa, indicates a potential association with alleviated oxidative stress and inflammation during myocardial I/R and thereby contributed to cardioprotection [49]. Collectively, these findings indicate that CYPs exert cardioprotective effects against myocardial I/R injury, at least in part, through potential modulation of gut microbiota composition, restoring intestinal microecological homeostasis, and reducing systemic inflammation through the gut–heart axis.

KEGG functional pathway analysis revealed that Metabolism was the dominant core pathway of gut microbiota in mice across all three groups, with Carbohydrate metabolism identified as the key secondary metabolic pathway. The I/R model induced a reduction in the abundance of the Carbohydrate metabolism pathway. Although CYPs intervention did not significantly upregulate this pathway, it enriched short-chain fatty acid-producing bacteria such as *Lachnospiraceae_NK4A136_group*, which may enhance the fermentation and utilization of carbohydrates by gut microbiota to generate short-chain fatty acids, thereby potentially exerting anti-inflammatory and antioxidant effects to alleviate myocardial I/R injury [49,50]. These probiotics ferment dietary carbohydrates to generate SCFAs, thereby exerting anti-inflammatory and antioxidant effects that alleviate myocardial I/R injury. Spearman correlation analysis further revealed significant associations between the gut microbiota and key indicators of myocardial injury. At the phylum level, this study demonstrated close relationships between the gut microbiota and host myocardial injury, oxidative stress, inflammation, and mitochondrial function. Bacillota was negatively correlated with tissue injury, oxidative stress, and inflammation and positively correlated with mitochondrial function, suggesting an association with a protective phenotype [51]. In contrast, *Thermodesulfobacteriota* showed the opposite pattern, suggesting a potential association with exacerbating oxidative stress and inflammation while impairing mitochondrial function [52]. *Verrucomicrobiota* and *Patescibacteria* were positively associated with antioxidant capacity and mitochondrial function and negatively with oxidative stress and inflammation, suggesting potential beneficial associations with redox balance, inflammation suppression, and mitochondrial homeostasis [53]. The genus-level correlation analysis further delineates associations between gut microbiota and host pathophysiology. *Unclassified_f_Lachnospiraceae* negatively correlated with markers of myocardial injury, oxidative stress, and inflammation and positively with energy metabolism and mitochondrial function, suggesting a potential protective association [54]. *Desulfovibrio* showed opposite correlations, indicating that its enrichment was associated with oxidative stress and inflammation, as well as impaired mitochondrial structure and function [55]. *Akkermansia* was positively associated with antioxidant capacity and mitochondrial function and negatively associated with oxidative stress and inflammation, supporting their potential associations with redox balance, inflammatory suppression, and mitochondrial homeostasis [56,57]. These findings suggest that CYPs may exert cardioprotective effects that are accompanied by alterations in the gut microbiota, potentially involving gut–heart communication. However, further mechanistic studies are needed to clarify whether these microbiota changes contribute causally to the observed cardioprotective effects. As microbiota-depletion experiments, fecal microbiota transplantation, and other microbiota-targeted interventions were not performed in the present study, a direct mechanistic role for the gut microbiota cannot yet be established.

## 5. Conclusions

**Figure 8:**
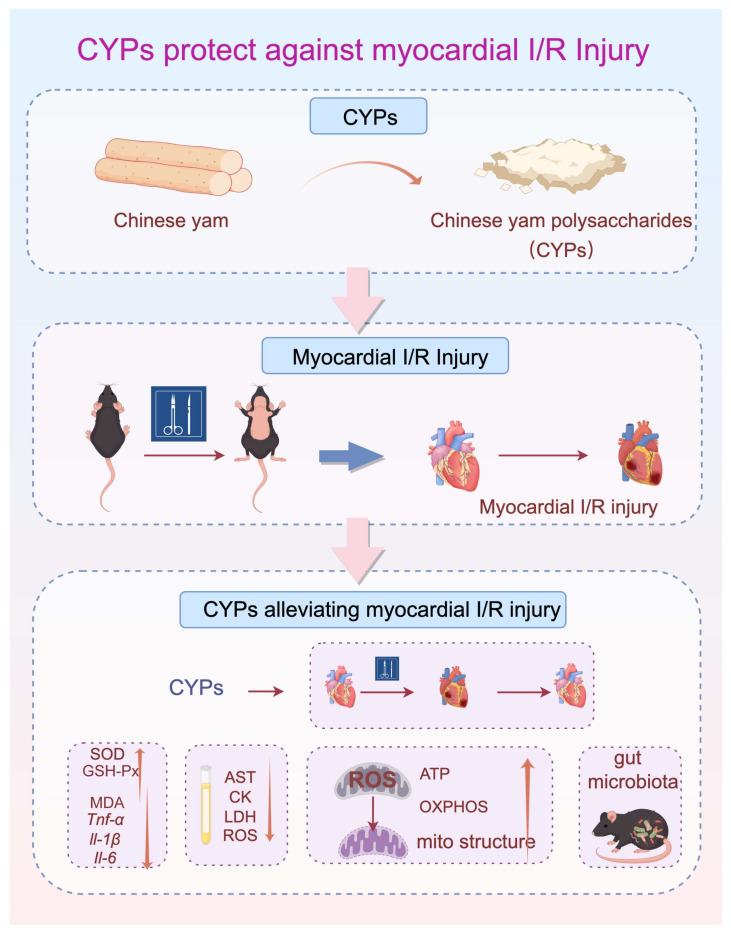
Proposed mechanisms by which CYPs may protect against myocardial I/R injury. Proposed schematic illustration of the potential mechanisms by which Chinese yam polysaccharides (CYPs) may protect against myocardial I/R injury through associations with reduced oxidative stress, inflammation, myocardial enzymes, and ROS; preservation of mitochondrial structure and ATP; and changes in gut microbiota. Abbreviations: CYPs, Chinese yam polysaccharides; I/R, ischemia/reperfusion; SOD, superoxide dismutase; GSH-Px, glutathione; MDA, malondialdehyde; TnI-α, cardiac troponin I-α; IL-1β, interleukin-1β; IL-6, interleukin-6; AST, aspartate aminotransferase; CK, creatine kinase; LDH, lactate dehydrogenase; ROS, reactive oxygen species; ATP, adenosine triphosphate; OXPHOS, oxidative phosphorylation. This figure was drawn using FigDraw (https://www.figdraw.com).

In summary, the present study demonstrates that CYPs exert significant cardioprotective effects against myocardial I/R injury through multiple targets and mechanisms. CYPs pretreatment effectively preserved cardiac function, reduced infarct size, and attenuated myocardial damage, as evidenced by decreased serum injury markers and oxidative stress indices. These protective effects were mediated through: (i) direct antioxidant and anti-inflammatory actions, as reflected by restored antioxidant enzyme activities, reduced ROS accumulation, and suppressed expression of pro-inflammatory cytokines; (ii) preservation of mitochondrial structural integrity and functional capacity, including normalized ultrastructure, enhanced ATP production, and upregulated expression of respiratory chain-related genes; and (iii) modulation of gut microbiota composition, characterized by the enrichment of beneficial genera with anti-inflammatory and antioxidant properties and the suppression of pathobionts associated with dysbiosis. Correlation analyses further demonstrated significant associations between CYP-regulated microbial taxa and improved cardiac metabolic phenotypes, suggesting that the gut–heart axis contributes substantially to the observed cardioprotective effects (Figure 8). Collectively, these findings identify CYPs as a promising functional food-derived candidate for adjunctive therapy in ischemic heart disease, with translational potential supported by their natural origin, favorable safety profile, and established use in dietary applications. However, it should be noted that CYPs were administered as a pretreatment in the present study, whereas clinical management of myocardial infarction is usually initiated after symptom onset rather than before ischemia. Future studies should focus on validating these preclinical findings in clinical settings, particularly by determining whether CYPs remain effective when administered after ischemia onset, during reperfusion, or in other clinically relevant therapeutic settings, and clarifying the precise causal relationships between specific gut microbial alterations and cardioprotection.

## Data Availability

The original contributions presented in this study are included in the article/Supplementary Material. Further inquiries can be directed to the corresponding authors.

## Supplementary Materials

The following supporting information can be downloaded at: https://www.mdpi.com/article/10.3390/nu18152464/s1. Table S1. Primer sequences used for RT-qPCR experiments.

## Abbreviations

The following abbreviations are used in this manuscript:

CYPs	Chinese Yam Polysaccharides
I/R	Ischemia/Reperfusion
LVEF	Left Ventricular Ejection Fraction
LVFS	Left Ventricular Fractional Shortening
LVID; s	Left Ventricular Internal Dimension at systole
LVESV	Left Ventricular End-Systolic Volume
CO	Cardiac Output
CI	Cardiac Index
AST	Aspartate Aminotransferase
CK	Creatine Kinase
CK-MB	Creatine Kinase-MB
LDH	Lactate Dehydrogenase
LDH-1	Lactate Dehydrogenase Isoenzyme 1
ROS	Reactive Oxygen Species
MDA	Malondialdehyde
SOD	Superoxide Dismutase
GSH-Px	Glutathione Peroxidase
Tnf-α	Tumor Necrosis Factor-α
Il-6	Interleukin-6
Il-1β	Interleukin-1β
ATP	Adenosine Triphosphate
TTC	Triphenyltetrazolium Chloride
TEM	Transmission Electron Microscopy
LEfSe	Linear Discriminant Analysis Effect Size
SCFA	Short-Chain Fatty Acid
Anp	Atrial Natriuretic Peptide
Bnp	Brain Natriuretic Peptide
