# Supplementary material for: Chinese Yam Polysaccharides Alleviate Myocardial Ischemia/Reperfusion Injury by Modulating Gut Microbiota, Restoring Mitochondrial Function, and Reducing Oxidative Stress
Source: Nutrients. 2026 Jul 28;18(15):2464. doi: 10.3390/nu18152464 (PMC13467936; doi:10.3390/nu18152464)
Supplement: Supplementary file 1 [file nutrients-18-02464-s001.zip › nutrients-4443409-supplementary.pdf]

Supplementary information

Table S1 Primer sequences used for RT-qPCR experiments

Gene	Primer	Sequence
Mouse *β-actin*	Forward	GTGACGTTGACATCCGTAAAGA
	Reverse	GCCGGACTCATCGTACTCC
Mouse *Il-1β*	Forward	TGCCACCTTTTGACAGTGATG
	Reverse	TGATGTGCTGCTGCGAGATT
Mouse *mt-Nd1*	Forward	TCCGAGCATCTTATCCACGC
	Reverse	GTATGGTGGTACTCCCGCTG
Mouse *mt-Nd4l*	Forward	ACCTCACCATAGCCTTCTCAC
	Reverse	TAGTCCTACAGCTGCTTCGC
Mouse *mt-Cyb*	Forward	ACGCAAACGGAGCCTCAATA
	Reverse	CCTCATGGAAGGACGTAGCC
Mouse *mt-COII*	Forward	ACCTGGTGAACTACGACTGC
	Reverse	GGACTGCTCATGAGTGGAGG
Mouse *mt-Atp6*	Forward	ACGCAAACGGAGCCTCAATA
	Reverse	CCTCATGGAAGGACGTAGCC
Mouse *Anp*	Forward	GCTTCCAGGCCATATTGGAG
	Reverse	GGGGGCATGACCTCATCTT
Mouse *Bnp*	Forward	GAGGTCACTCCTATCCTCTGG
	Reverse	GCCATTTCCTCCGACTTTTCTC
Mouse *Tnf- α*	Forward	ATGGCCTCCCTCTCATCAGT
	Reverse	TTTGCTACGACGTGGGCTAC
Mouse *ll-6*	Forward	CCCCAATTTCCAATGCTCTCC
	Reverse	CGCACTAGGTTTGCCGAGTA
